# Development of rapid and selective epoxidation of α-pinene using single-step addition of H_2_O_2_ in an organic solvent-free process

**DOI:** 10.1039/d1ra05940h

**Published:** 2021-10-07

**Authors:** Mohamad Faiz Mukhtar Gunam Resul, Abdul Rehman, Ana María López Fernández, Valentine C. Eze, Adam P. Harvey

**Affiliations:** School of Engineering, Newcastle University Newcastle upon Tyne NE1 7RU UK; Department of Chemical and Environmental Engineering, Faculty of Engineering, Universiti Putra Malaysia 43400 UPM Serdang Selangor Malaysia; Department of Chemical and Polymer Engineering, University of Engineering and Technology Lahore Faisalabad Campus Pakistan a.rehman2@uet.edu.pk

## Abstract

This study reports substantial improvement in the process for oxidising α-pinene, using environmentally friendly H_2_O_2_ at high atom economy (∼93%) and selectivity to α-pinene oxide (100%). The epoxidation of α-pinene with H_2_O_2_ was catalysed by tungsten-based polyoxometalates without any solvent. The variables in the screening parameters were temperatures (30–70 °C), oxidant amount (100–200 mol%), acid concentrations (0.02–0.09 M) and solvent types (*i.e.*, 1,2-dichloroethane, toluene, *p*-cymene and acetonitrile). Screening the process parameters revealed that almost 100% selective epoxidation of α-pinene to α-pinene oxide was possible with negligible side product formation within a short reaction time (∼20 min), using process conditions of a 50 °C temperature in the absence of solvent and α-pinene/H_2_O_2_/catalyst molar ratio of 5 : 1 : 0.01. A kinetic investigation showed that the reaction was first-order for α-pinene and catalyst concentration, and a fractional order (∼0.5) for H_2_O_2_ concentration. The activation energy (*E*_a_) for the epoxidation of α-pinene was ∼35 kJ mol^−1^. The advantages of the epoxidation reported here are that the reaction could be performed isothermally in an organic solvent-free environment to enhance the reaction rate, achieving nearly 100% selectivity to α-pinene oxide.

## Abstract 2

Products obtained from the oxidation of α-pinene with hydrogen peroxide (H_2_O_2_) in the presence of tungsten-based polyoxometalates (α-pinene 1, α-pinene oxide 2, pinanediol 3, campholenic aldehyde 4, sobrerol 5, verbenol 6 and verbenone 7).

## Graphical abstract

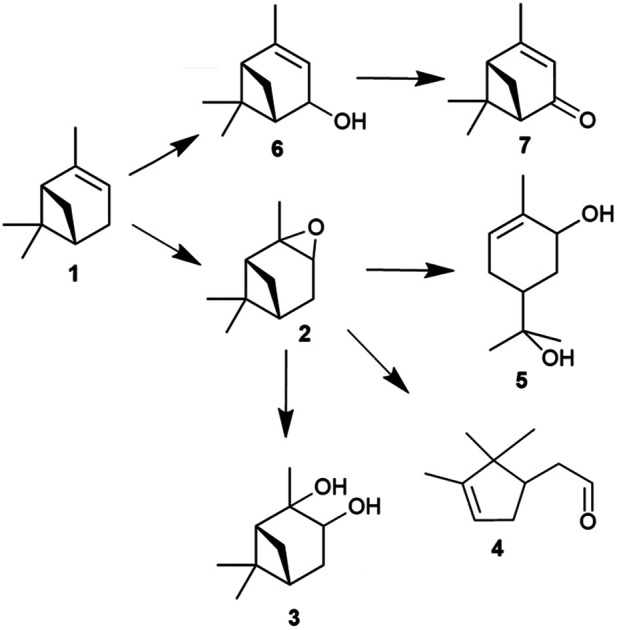

## 1 Introduction

**Fig. 1:**
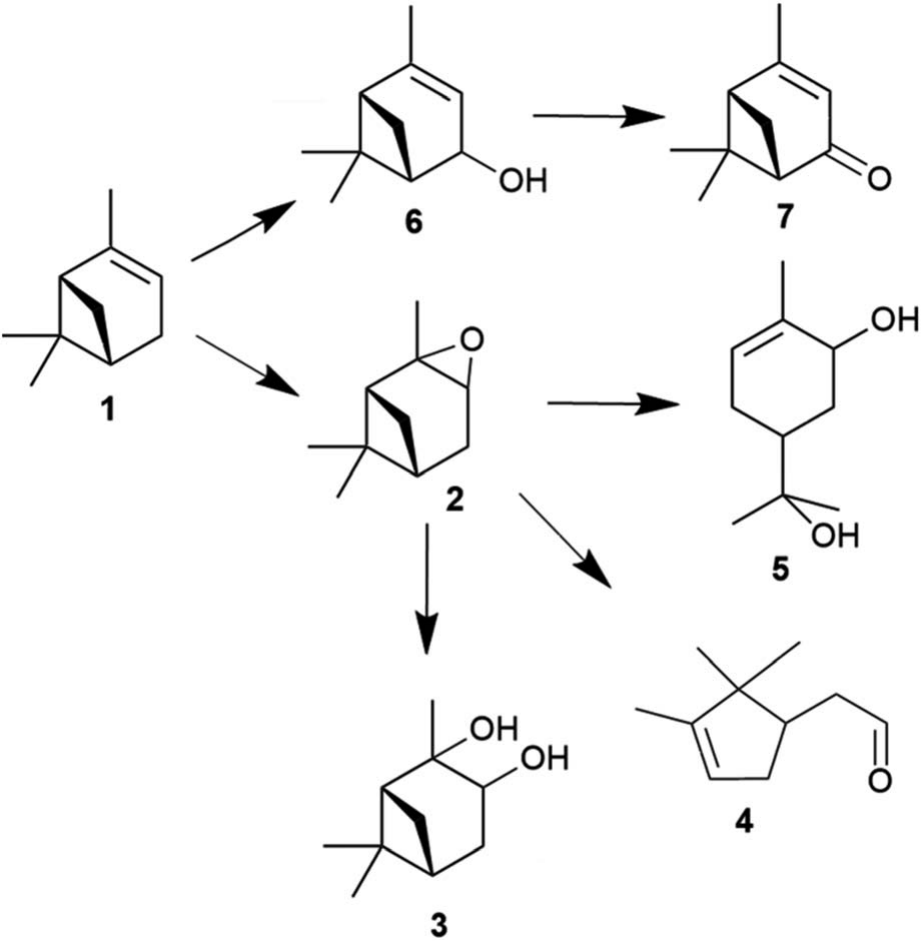
Products obtained from the epoxidation of α-pinene with hydrogen peroxide (H_2_O_2_) catalysed by tungsten-based polyoxometalates; α-pinene 1, α-pinene oxide 2, pinanediol 3, campholenic aldehyde 4, sobrerol 5, verbenol 6 and verbenone 7.

Waste biomass-derived terpenes such as α-pinene are important sources of flavours, fragrances and pharmaceutical applications.^1–7^ α-Pinene is a central component of turpentine oil, a useful by-product of wood and paper industries. The sustainable utilisation of waste biomass-derived α-pinene is beneficial, as it decreases waste and maintains CO_2_ neutrality.^8^ The oxidation of α-pinene generates many valuable products (Fig. 1), such as α-pinene oxide, verbenol and verbenone,^9,10^ the last of which is an important intermediate in many processes and has many applications.^11^ The isomerisation of α-pinene oxide into products for instance campholenic aldehyde is useful for the production of fragrances.^12^

The epoxidation of α-pinene (1) with H_2_O_2_ using tungsten-based polyoxometalates produced α-pinene oxide (2) as the main product, as the oxidant is attracted to the more-substituted π bonds. In the presence of H^+^ and H_2_O, the α-pinene oxide can undergo hydrolytic decomposition to form pinanediol (3) and sobrerol (5). α-Pinene oxide can also rearrange to form campholenic aldehyde (4) and experience allylic oxidation to produce verbenol (6), which can be further transformed to verbenone (7) by oxidative dehydrogenation. The dominance of epoxidation or allylic oxidation is due to the nature of the catalyst and the generation of radicals during the reaction.^13^ In principle, an active catalyst could also abstract H atoms from more than one position, and various hydroperoxides could be formed as intermediates, such as verbenyl hydroperoxides.^14,15^ In addition, α-pinene could isomerise to form many products, including β-pinene, limonene, terpineol, camphene and 3-carene.^16–18^ Many products, such as pinocarveol, isopinocamphone and *trans*-carveol, have been formed from the rearrangement process of α-pinene oxide.^12,19^ Moreover, polymeric compounds can also be formed during the oxidation process.

In recent decades, there is a lot of research in the epoxidation of α-pinene with hydrogen peroxide (H_2_O_2_).^20–30^ H_2_O_2_ is favoured over many types of oxidants, as it only yields H_2_O as a by-product.^31,32^ Developing a selective process for the epoxidation of α-pinene is challenging due to the tendency of α-pinene and its products to undergo rearrangement, hydrolysis, double bond migration and over-oxidation.^33,34^ The exothermic reaction usually necessitates the use of an excessive solvent or the drop-wise inclusion of an oxidant to mitigate the exotherm. This increases the reaction time, which in turn increases reactor size and consequently the capital and running costs. Among the several types of catalysts used for this reaction, a polytungstophosphate is effective with H_2_O_2_.^35–39^

In our previous work, we have developed a 100% selective method of limonene epoxidation with H_2_O_2_ in an organic solvent-free condition.^40^ In this work, we studied the possibility of developing a highly selective process using a more challenging terpene *i.e.*, α-pinene. Hence, this work aimed to improve/intensify the α-pinene epoxidation process by removing the need for an organic solvent and to develop a process with 100% selectivity towards α-pinene oxide. This work explores the various conditions that affect the formation of many side products in α-pinene epoxidation. A kinetic study was performed to formulate an analytical kinetic model for the α-pinene epoxidation. The possibility of reducing reaction time while maintaining an isothermal condition was also studied.

## 2 Experimental

### 2.1 Materials

Following reagents were utilised in this study: tungstic acid sodium salt dihydrate (Na_2_WO_4_·2H_2_O), hydrogen peroxide (H_2_O_2_) (30% wt in H_2_O), phosphoric acid (H_3_PO_4_) 42.5%, sulphuric acid (H_2_SO_4_) 48.5%, 0.02 M potassium permanganate (KMNO_4_) solution (0.1 N), anhydrous sodium sulphate (Na_2_SO_4_, ≥99%), anhydrous sodium chloride (NaCl, ≥99%), α-pinene (98%), *p*-cymene (99%), acetonitrile (99%), toluene (99%), 1,2-dichloroethane (99%), Adogen 464® phase transfer catalyst. α-Pinene oxide (97%), pinanediol (99%), *trans*-sobrerol (99%), campholenic aldehyde (≥96%), verbenol (95%) and verbenone (≥99%) were used for product identification. All reagents were purchased from Merck, UK.

### 2.2 Preparation of the oxidant

The polytungstophosphate catalyst ({PO_4_[WO(O_2_)_2_]_4_^3–^) was prepared following an existing procedure.^41^ A tungsten source, Na_2_WO_4_·2H_2_O (0.4 g), was first added to a stirred H_2_O_2_ (30% wt, 122 mmol) solution at the desired temperature, typically 50 °C. A phosphate source, H_3_PO_4_ (42.5%, 0.06 mmol), was then added to the well-mixed solution, and the pH of the solution was adjusted to <1.0 with H_2_SO_4_ (48.5%, 4 mmol). A phase transfer catalyst, Na_2_SO_4_ (5.2 g), was later added and continuously stirred until fully dissolved. The addition of a saturated amount of Na_2_SO_4_ for the epoxidation of terpenes with H_2_O_2_ has been investigated in our previous work and was found to significantly inhibit hydrolysis of epoxides and thus increasing its selectivity.^40^

### 2.3 Epoxidation of α-pinene

A typical procedure used in this study for the epoxidation reaction is as follows: α-pinene (122 mmol) and Adogen 464® (1 g) as a phase transfer catalyst, were first added to a jacketed 150 mL flask. Solvent (100–500 mol%) was added to achieve isothermal conditions. The mixture was stirred vigorously (1250 rpm) at the desired temperature (30–70 °C). Temperature control was provided by a water bath (VWR MX7LR-20) and a thermocouple. The prepared oxidant (12.5 mL) was later added to the jacketed flask and allowed to react. About 1 mL of samples were collected by the reaction at selected periods of 5–120 min. The samples were analysed for α-pinene oxide content using a gas chromatography method. For purification, the organic phase was isolated from the aqueous phase using a separating funnel after the reaction was completed. To destabilise the phase transfer catalyst, 5 mL of 0.1 M NaCl was added with vigorous shaking before the phases were separated. The bulk organic layer obtained after the completion of the reaction was dried with anhydrous Na_2_SO_4_, and the unreacted α-pinene was separated from α-pinene oxide using a rotary evaporator coupled with a silicone oil bath at 150 °C and a reduced pressure of less than 50 mbar. The purity of the α-pinene oxide achieved after removal of unreacted α-pinene was ≥98%. All reactions performed were ensured to be conducted similarly with very minimal variations between the reactions. The same set of apparatus, heaters and stirrers were used to minimise the probability of other influencing factors.

### 2.4 Kinetics of epoxidation of α-pinene and effects of reaction parameters

In this study, the kinetic parameters and reaction order was determined using an initial-rate method on the basis of pseudo-first-order conditions. The rate laws for all the reactions considered are shown in eqn (1)–(3), *r*_decomp_, *r*_oxidative_ and *r*_epox_ are the rates of reaction for the H_2_O_2_ decomposition, the formation of oxidative species and α-pinene epoxidation, respectively, while *k*_decomp_, *k*_oxidative_ and *k*_epox_ are the rate constants for each rate law. The reaction order for the catalyst was studied using Na_2_WO_4_·2H_2_O concentrations in the range of 0.008–0.016 M, and fixed initial concentrations of α-pinene (1.25 M), H_2_O_2_ (1.25 M) and 500 mol% solvent (toluene). The study was performed using a lower concentration of catalyst (≤0.016 M), since higher metal loading is known to cause H_2_O_2_ decomposition.^42^ The reaction order for α-pinene was determined by varying the initial concentration of α-pinene from 0.25–1.25 M, while keeping the concentrations of H_2_O_2_ (1.25 M) and Na_2_WO_4_ (0.012 M) constant and in excess. Similarly, the reaction order for H_2_O_2_ was established by changing the initial concentration of H_2_O_2_ from 0.25–1.25 M, with the initial concentration of α-pinene (1.25 M) and Na_2_WO_4_ (0.012 M) being kept constant and in excess.

1 *r*_decomp_ = *k*_decomp_[H_2_O_2_]

2 *r*_oxidative_ = *k*_oxidative_[H^+^]([8H_2_O_2_][4WO_4_^2−^][PO_4_^3−^][3Q] − *K*_eq_^−1^[Q_3_POM][7H_2_O])

3 *r*_epox_ = *k*_epox_[pinene][Q_3_POM]

Effects of temperatures, oxidant and H^+^ concentrations, and solvent types on the epoxidation of α-pinene were also studied. The reaction temperatures in the range of 30–70 °C were studied, whereas the effect of oxidant amount on the α-pinene epoxidation was carried out by changing the quantity of H_2_O_2_ from 100–200 mol% for α-pinene. The effects of H^+^ on the epoxidation of α-pinene was investigated by varying the concentration of H_2_SO_4_ from 0.04–0.09 M, with the initial pH ≤ 1 in all cases as measured using a pH meter. The tungsten-based polyoxometalates were reported to be stable in an acidic environment.^34,35,41,43,44^ However, an acid-labile epoxide such as α-pinene oxide could undergo some competitive reactions in such an environment, hence the need for further investigations. The exothermic nature of the α-pinene epoxidation reaction with H_2_O_2_ is typically mitigated by the drop-wise addition of H_2_O_2_ and after adding an organic solvent. To mitigate the exothermic nature of the α-pinene epoxidation reaction with H_2_O_2_, a selection of organic solvents: 1,2-dichloroethane, toluene, *p*-cymene and acetonitrile were investigated for this application in the ranges of 100–500 mol% based on the α-pinene.

### 2.5 Product analysis (gas chromatography)

The product was analysed using a 5890 Hewlett Packard Series II gas chromatography (GC) provided with a CP Wax Capillary column (BPX70). About 40 μL of each sample was collected into 2 mL vials and mixed with 1.96 mL of chloroform. Naphthalene (10 mg) was used as an internal standard. About 0.5 μL of the product collected from the reaction was injected into the GC by an auto-sampler. The GC oven temperature was set as: an initial temperature of 80 °C was maintained for 4 min. The temperature was then ramped up by 15 °C min^−1^ to 260 °C, which was maintained for 10 min. The total running time was approximately 26 min per sample. The GC injector and flame ionisation detector (FID) temperatures were both set at 250 °C. The reaction conversions and yields were determined by GC analysis, following internal standard response factors obtained from calibration data using the commercial analytical grade α-pinene, α-pinene oxide, pinanediol, *trans*-sobrerol, campholenic aldehyde, verbenol and verbenone. The response factors were obtained by the plots of ratios of the GC peak areas of each analyte to the internal standard compared to mass ratios of the analyte. The GC analysis for all the analytes showed less than ∼2% error for the mean values taken from parallel experiments.

The α-pinene conversion was determined using the expression in eqn (4), where *C*_0_ is the initial concentration of α-pinene, and *C*_i_ is the concentration of α-pinene in the sample. When H_2_O_2_ is the limiting reactant, the concentration of H_2_O_2_ and its conversion were determined by titration with 0.02 M potassium permanganate solution. The product yields were calculated using eqn (5), where *X*_i_ is the concentration of specific products in the sample as determined by GC.4
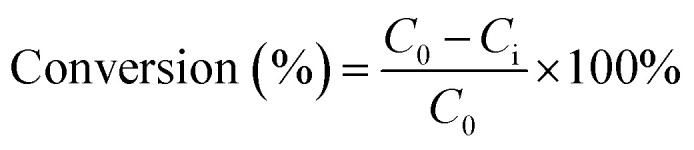
5
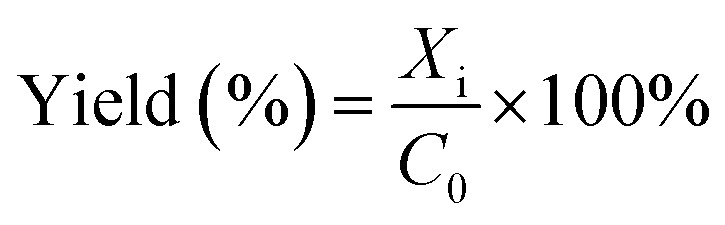


## 3 Results and discussion

### 3.1 Kinetics study

**Fig. 2:**
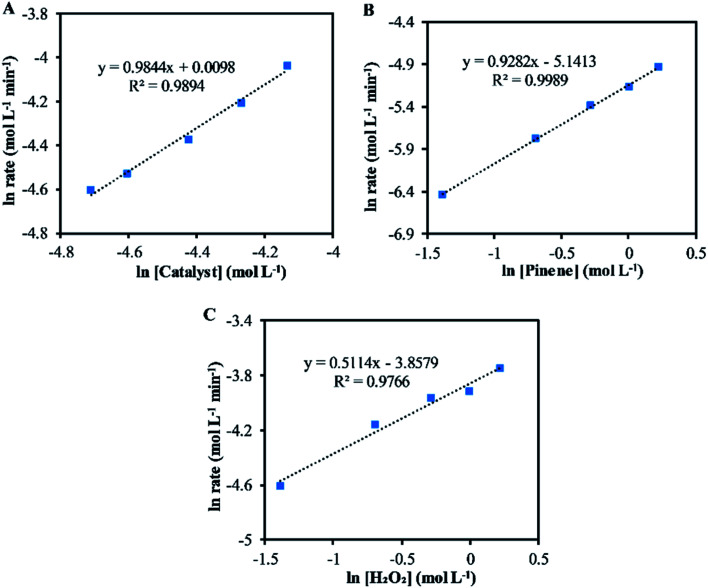
The plot of the natural log between the initial reaction rate and (A) catalyst concentration (B) α-pinene concentration (C) H_2_O_2_ concentration.

Fig. 2 shows the results for the investigations of the reaction orders with respect to the catalyst, α-pinene and H_2_O_2_. The natural log graph between the initial rates and the catalyst concentrations gives a straight line having a gradient of ∼1, showing that the first order in terms of catalyst concentration (Fig. 2A). Similarly, the gradient obtained from the natural log graph between the initial rates and the α-pinene concentration confirms a first-order reaction (Fig. 2B). Fig. 2C shows that the gradient for the natural log graph between the initial rates and the H_2_O_2_ concentration was ∼0.5, which indicates a stepwise process where H_2_O_2_ will form the peroxo species with the catalyst precursor, tungstate (WO_4_^2−^) and phosphate (PO_4_^3−^). The peroxo species in turn oxidise the double bonds to form epoxides. Therefore, according to eqn (2), increasing the concentration of H_2_O_2_ increases the formation of the active peroxo species, but this is limited by the concentration of WO_4_^2−^ and PO_4_^3−^. Since the concentration of both WO_4_^2−^ and PO_4_^3−^ is constant, increasing the amount of H_2_O_2_ does not cause a first-order kinetic reaction.

### 3.2 Effect of temperature

**Fig. 3:**
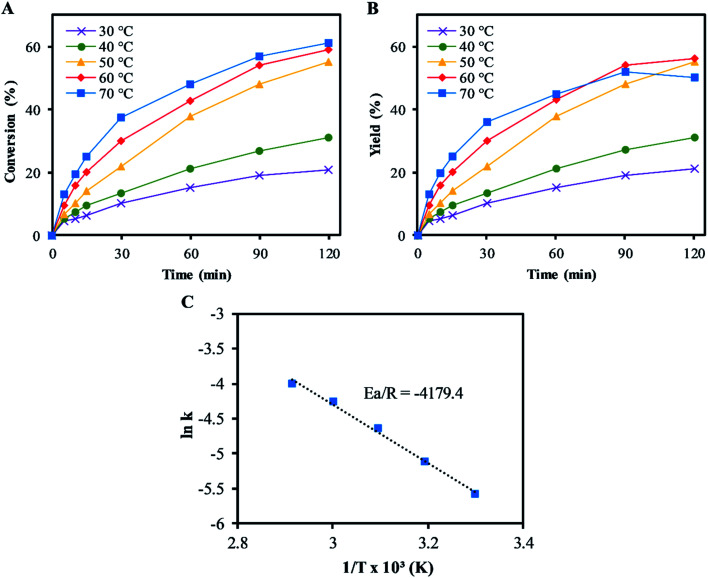
Effect of change in temperature on α-pinene epoxidation: (A) conversion of α-pinene; (B) yield of α-pinene oxide. (Reaction conditions: α-pinene (1.25 M), H_2_O_2_ (1.25 M), Na_2_WO_4_·2H_2_O (0.012 M), H_2_SO_4_ (0.05 M) and toluene (500 mol%); (C) determination of Arrhenius activation energy for α-pinene epoxidation. The lines represent the trends between data points.

Fig. 3 shows the effects of reaction on the epoxidation of α-pinene. The rates of conversions of α-pinene increased with temperatures from 30–70 °C, as shown in Fig. 3A. This trend was also followed by the α-pinene oxide yields, except at 70 °C, where a reduction in the α-pinene oxide yield was observed after 60 min reaction time. The reduction in the yield was due to the α-pinene oxide undergoing both hydrolytic decomposition and rearrangement, resulting in the formation of side products: mainly sobrerol (∼3%) and campholenic aldehyde (∼1%). Allylic oxidation becomes significant at temperatures higher than 60 °C, where the yield of verbenol is ∼5% (negligible verbenone). While higher temperatures increase the rate of α-pinene conversion, they reduce selectivity to α-pinene oxide. At temperatures below 50 °C, the process was highly selective to α-pinene oxide, with little to no formation of any side products. Hence this temperature was used in all subsequent experiments, as the highest temperature at which no side products were detected.

The Arrhenius activation energy (*E*_a_) for the epoxidation was determined to be ∼35 kJ mol^−1^, using the gradient of the plot of ln(*k*_epox_) *versus* 1/*T* in Fig. 3C, which is not far from ∼40 kJ mol^−1^ reported by Becerra *et al.*^13^ and lower than that suggested by Cánepa *et al.* (∼68 kJ mol^−1^) for the epoxidation of α-pinene.^42^ The lower activation energy was obtained from this study compared to ∼68 kJ mol^−1^ in Cánepa *et al.* when a heterogeneous catalyst was used,^42^ indicating a faster epoxidation reaction rate in homogeneous catalysis. The rate constant, *k*_epox_ at 50 °C was determined to be 9.2 × 10^−3^ L mol^−1^ s^−1^.

### 3.3 Effect of oxidant amount

**Fig. 4:**
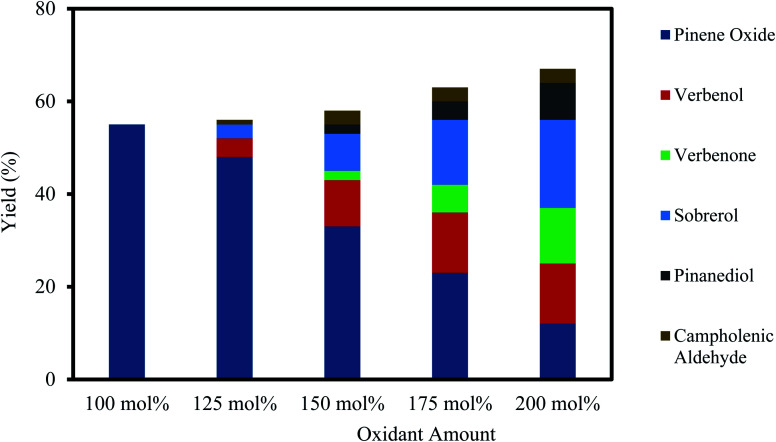
Effect of the oxidant amount on α-pinene epoxidation and the distribution of the products. Reaction conditions: temperature (50 °C), reaction time (120 min), α-pinene (1.25 M), Na_2_WO_4_·2H_2_O (0.012 M), H_2_SO_4_ (0.05 M) and toluene (500 mol%).

Results of the H_2_O_2_ oxidant amount on the epoxidation of α-pinene at the concentrations of 100–200 mol% are shown in Fig. 4. As depicted in Fig. 4, the selectivity to α-pinene oxide decreased by increasing the oxidant amount. The yields of α-pinene oxide were 55% and 12% for the oxidant amounts of 100 and 200 mol%, respectively. At a greater-than-equimolar amount of oxidant, epoxidation and allylic oxidation occur simultaneously.^45^ The yield of verbenol increases from 4–13% with an increase in oxidant amount from 125–200 mol%, which subsequently increases the yield of verbenone. These results are in accord with an existing study, which states that an increase in H_2_O_2_ concentration generates more radicals and favours the allylic oxidation route, which in turn oxidises verbenol to verbenone.^46^

As shown in Fig. 4, a higher oxidant amount also causes a lower yield of α-pinene oxide, as the rate of hydrolytic decomposition increases, which results in the formation of sobrerol and pinanediol. The increased rate of hydrolytic decomposition is mainly influenced by the increasing concentration of H_2_O, which is proportionate to the oxidant amount, as 30% (wt/wt) H_2_O_2_ solution was used. The yield of sobrerol rose from 3–19% when the oxidant amount is increased from 125–200 mol%. Pinanediol was observed between an oxidant amount of 150 and 200 mol%, with its yield increasing from 2–8%. A small amount of campholenic aldehyde (∼3%) is always present in the reaction mixture at higher oxidant amounts but does not correlate to the increasing amount of oxidant. It appears that increasing amounts of oxidant have little effect on the transformation of α-pinene oxide to campholenic aldehyde. To maintain the highest selectivity towards α-pinene oxide, an equimolar amount of oxidant and α-pinene is recommended.

### 3.4 Effect of acid concentration

**Fig. 5:**
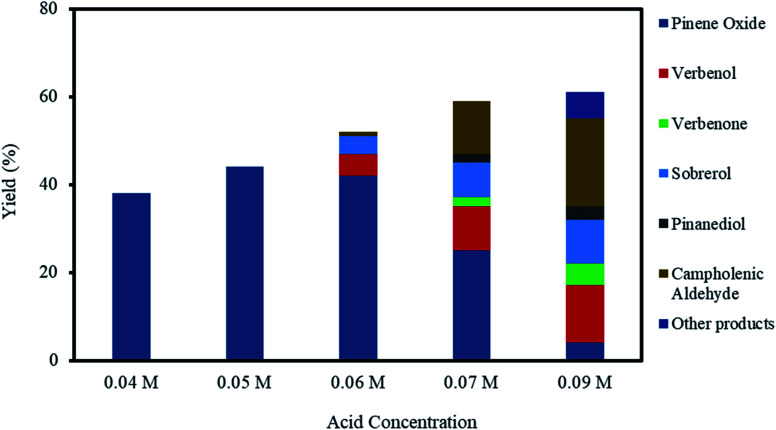
Effect of acid concentration on α-pinene epoxidation and distribution of the products. Reaction conditions: temperature (50 °C), reaction time (120 min), α-pinene (1.25 M), H_2_O_2_ (1.25 M), Na_2_WO_4_·2H_2_O (0.012 M) and toluene (500 mol%).

The effect of acid concentration in the reaction media was investigated by varying the H_2_SO_4_ concentrations from 0.04–0.09 M, as shown in Fig. 5, for the investigations using H_2_SO_4_ concentrations from 0.04–0.09 M. The selectivity to α-pinene oxide decrease dramatically with an increasing acid concentration, for example, the yields were 38% at 0.04 M and 4% at 0.09 M acid concentrations.

At acid concentrations of 0.06 M and higher, the decrease in the final yield of α-pinene oxide is primarily due to the significant increase in α-pinene oxide rearrangement; α-pinene oxide was progressively consumed to an almost complete conversion, mainly forming campholenic aldehyde. The yield of campholenic aldehyde rises from ∼1 to ∼20% with increasing acid concentrations. Furthermore, at an acid concentration of 0.09 M, other products consist of mainly aldehyde and alcohol were detected by the analytical methods with a total calculated yield of 6%. These observations are largely influenced by the increase in H^+^ concentration, which acts as a Lewis acid. The H^+^ reacts with the oxygen atoms in α-pinene oxide, causing the C–C bonds to split and form aldehyde and alcohol.^12,45^

The decrease in α-pinene oxide yield is also due to the increase in the hydrolytic decomposition of the oxide. The yield of sobrerol increases from 4–10% with the increasing acid concentration. On the other hand, the yield of pinanediol was only 2 and 3% at acid concentrations of 0.07 and 0.09 M, respectively. The hydrolytic decomposition of α-pinene oxide becomes less prevalent with increasing acid concentrations than with increases in the oxidant amount, as discussed in Section 3.3. This confirms the need for high H_2_O concentrations to degrade α-pinene oxide through hydrolysis, while increasing acid (H^+^) concentrations favour the rearrangement process.

The competing allylic oxidation is apparent when acid concentration is higher than 0.06 M, with the yield of verbenol increasing from 5–13%. Verbenone was only observed at acid concentrations of 0.07 and 0.09 M, with yields of ∼2 and 5%, respectively. From these results, it can be deduced that the epoxidation route is more kinetically favoured than allylic oxidation, even when the acid concentration is increased, as confirmed by the increasing total yield of α-pinene oxide rearrangement and hydrolysis products as compared to allylic oxidation products.

The epoxidation of α-pinene is only 100% selective to α-pinene oxide when the acid concentration is below 0.05 M. The pH of the aqueous phase increases throughout the reaction time mainly caused by the formation of H_2_O as H_2_O_2_ was consumed. At acid concentrations of 0.06 M and higher, the final pH was less than 2, while acid concentrations lower than 0.05 M resulted in a final pH of about ∼2.5. It is presumed that higher acid concentrations not only destabilise the peroxo species but also reduce H^+^ concentration, which could affect the selectivity to α-pinene oxide. In this regard, the use of a pH buffer might favour the acid-catalysed formation of side products.

The reaction having a 0.05 M acid concentration at 70 °C (Section 3.2) did not yield as many side products as the reaction with a 0.09 M acid concentration at 50 °C. This highlights the importance of acid concentration to this type of epoxidation, which holds the key to developing a highly selective epoxidation of α-pinene. It is worth mentioning that a control experiment was carried out in the absence of an acid. This raised the pH of the aqueous peroxide solution above 4, which presumably retarded the formation of the active peroxo species. No conversion of α-pinene to any product was observed during the reaction time.

### 3.5 Effects of organic solvents

**Fig. 6:**
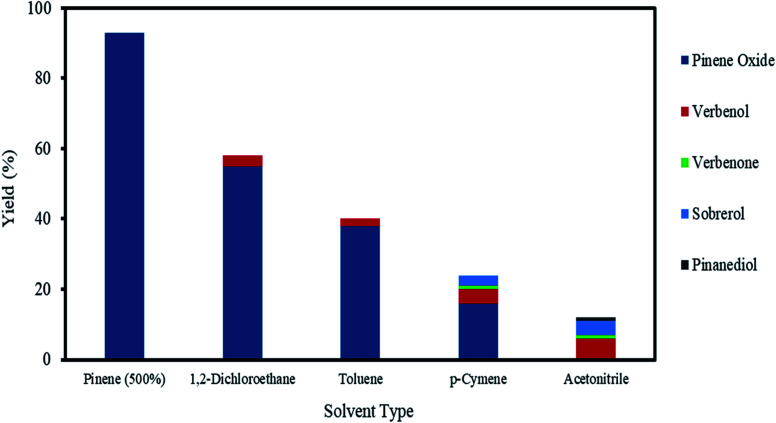
The effects of solvents on α-pinene epoxidation. Reaction conditions: temperature (50 °C), reaction time (120 min), α-pinene (1.25 M), H_2_O_2_ (1.25 M), Na_2_WO_4_·2H_2_O (0.012 M), H_2_SO_4_ (0.05 M) and solvent amount (500 mol%) for each solvent.

The exothermic nature of the α-pinene epoxidation with H_2_O_2_ is typically mitigated by the drop-wise addition of H_2_O_2_ and by the addition of an organic solvent. Therefore, a selection of solvents, from polar to non-polar, in addition to an organic solvent-free system using α-pinene in a higher molar ratio (>200 mol%) were investigated as shown in Fig. 6.

As shown in Fig. 6, in the presence of various solvents, the conversion of α-pinene decreases in the following order: 1,2-dichloroethane (58%) > toluene (40%) > *p*-cymene (24%) > acetonitrile (12%). Of all the solvents, 1,2-dichloroethane improved the conversion of α-pinene epoxidation most. Under these conditions, the yield of α-pinene oxide is 55%, with only verbenol (3%) being observed as a side product. Toluene afforded yields of α-pinene oxide and verbenol of about 38 and 2%, respectively. The conversion of α-pinene in *p*-cymene as a solvent after a reaction time of 120 min was about 24%. *p*-Cymene was less selective to α-pinene oxide than toluene and 1,2-dichloroethane. The conversion of α-pinene is the lowest when acetonitrile is used, with no α-pinene oxide being detected within a reaction time of 120 min. This was due to the complete conversion of α-pinene oxide to its hydrolysis products, sobrerol (4%) and pinanediol (∼1%). Allylic oxidation is more prevalent in acetonitrile, in which the yields of verbenol and verbenone are 6 and ∼1%, respectively. This is consistent with an existing study showing that allylic oxidation is favoured when acetonitrile is used.^47^ There was no formation of campholenic aldehyde observed with any of the solvents used. This can be attributed to the lower acid concentration (0.05 M), which hampered the rearrangement of α-pinene oxide as explained in Section 3.4.

**Fig. 7:**
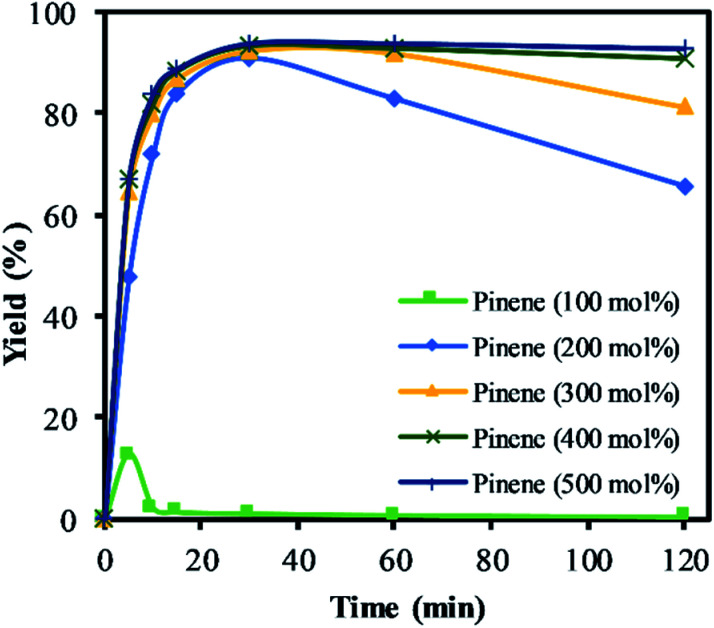
The yield of α-pinene oxide *vs.* time based on the amount of α-pinene. Reaction conditions: temperature (50 °C), H_2_O_2_ (1.25 M), Na_2_WO_4_ (0.012 M) and H_2_SO_4_ (0.05 M). The lines represent the trends between data points.

The highest yield and selectivity to α-pinene oxide was obtained when α-pinene was used without any additional organic solvent in a higher molar ratio than H_2_O_2_ (Fig. 7). The calculated conversion and yield were determined on the basis of H_2_O_2_ as the limiting reactant. Fig. 7 shows the increasing yield of α-pinene oxide when the amount of α-pinene is increased to 500 mol%. In the absence of an organic solvent and with an amount of α-pinene equimolar to H_2_O_2_ (100 mol%), the yield of α-pinene oxide reached its maximum (12%) before completely converting to various side products. Interestingly, the mass balance shows that the conversion of H_2_O_2_ does not account for all the products detected by GC. It is presumed that an oligomeric and polymeric compound was formed throughout the reaction time, assisted by the exothermicity. This became evident when a viscous layer of liquid was observed in the organic phase, justifying the need for a solvent in such processes.

The yield of α-pinene oxide improves significantly when α-pinene was used in a higher molar ratio (>200 mol%), to double up as solvent. Maximum yield was reached within 20 min of the reaction time due to a high reaction rate. The yields of α-pinene oxide at 200 and 300 mol% reached a maximum of 91 and 93%, respectively. However, the selectivity to α-pinene oxide decreases gradually over time due to the combination of the hydrolytic decomposition and rearrangement processes. Further increasing the amount of α-pinene up to 500 mol% improves the selectivity of α-pinene oxide and results in isothermal conditions. With 500 mol% of α-pinene, the yield of α-pinene oxide reaches 93%, with 100% selectivity to α-pinene oxide throughout the reaction. This result can be illustrated by the rapid formation of α-pinene oxide, which fully utilised the oxidant in the reaction. This is confirmed by the titration of H_2_O_2_. The rapid consumption of active oxygen, coupled with the lower acid concentration, significantly reduces the propagation of radicals. This, in turn, suppresses the allylic oxidation and favours epoxidation as the dominant route; at α-pinene amounts of 200 mol% and higher, little to no formation of allylic products was observed. The large reservoir of α-pinene not only helps to mitigate the exotherm but also to isolate α-pinene oxide in the organic phase, minimising its contact with H^+^ and H_2_O presents in the aqueous phase in this biphasic reaction.

The easy separation of the organic phase and the subsequent separation of α-pinene from α-pinene oxide in a vacuum evaporator allows the unreacted α-pinene to be recycled and reused. This results in a rapid process whereby α-pinene is converted to α-pinene oxide with high selectivity in an organic solvent-free environment within a short reaction time (∼20 min). The reduced reaction time is achieved by adding the oxidant with a single-step addition. Catalyst recycling should be considered to further improve the process, making it industrially and environmentally feasible.

## 4 Conclusions

The epoxidation of α-pinene typically leads to many side products and is usually performed in excess solvent or using drop-wise addition of the oxidant, which reduces the reaction rates and lengthens the reaction time, while the use of solvent causes additional separation step. In this work, 93% conversion of H_2_O_2_ with 100% selectivity to α-pinene oxide within 20 min reaction time was achieved using a single-step addition of the oxidant without an organic solvent. It was demonstrated that a low concentration of acid (∼0.05 M) should be used, as this prevented the formation of various side products. The selective process was able to be achieved through the rapid and efficient consumption of oxidants by increasing the substrate molar ratio in the absence of an organic solvent and lowering the concentration of acid to allow a predominantly epoxidation reaction route. This study also found that increasing the amount of oxidant increases the rate of allylic oxidation. Reaction kinetics revealed that the reaction is first order for α-pinene and the catalyst, and a reaction order of 0.5 for the H_2_O_2_. The activation energy for the α-pinene epoxidation was determined to be 35 kJ mol^−1^.

## Conflicts of interest

There are no conflicts to declare.

## Supplementary Material
